# Oleuropein, a Bioactive Compound from *Olea europaea* L., as a Potential Preventive and Therapeutic Agent in Non-Communicable Diseases

**DOI:** 10.3390/antiox8120578

**Published:** 2019-11-22

**Authors:** Chiara Nediani, Jessica Ruzzolini, Annalisa Romani, Lido Calorini

**Affiliations:** 1 Department of Experimental and Clinical Biomedical Sciences “Mario Serio”, University of Florence, viale Morgagni 50, 50134 Florence, Italy; jessica.ruzzolini@unifi.it (J.R.); lido.calorini@unifi.it (L.C.); 2 PHYTOLAB (Pharmaceutical, Cosmetic, Food Supplement, Technology and Analysis)-DiSIA, University of Florence, Via U. Schiff, 6, 50019 Sesto Fiorentino, Florence, Italy; annalisa.romani@unifi.it; 3 Istituto Toscano Tumori and Center of Excellence for Research, Transfer and High Education (DENOTHE), University of Florence, Piazza di San Marco 4, 50121 Florence, Italy

**Keywords:** *Olea europaea* L., oleuropein, extra-virgin olive oil, health effects, non-communicable diseases, oxidative stress, inflammation, autophagy, amyloid

## Abstract

Growing scientific literature data suggest that the intake of natural bioactive compounds plays a critical role in preventing or reducing the occurrence of human chronic non-communicable diseases (NCDs). Oleuropein, the main phenolic component of *Olea europaea* L., has attracted scientific attention for its several health beneficial properties such as antioxidant, anti-inflammatory, cardio- and neuro-protective, and anti-cancer. This article is a narrative review focused on the current literature concerning the effect of oleuropein in NCDs, such as neuro- and cardiovascular diseases, diabetes mellitus, chronic kidney diseases, and cancer, by its putative antioxidant and anti-inflammatory activity, but also for its other peculiar actions such as an autophagy inducer and amyloid fibril growth inhibitor and, finally, for its anti-cancer effect. Despite the increasing number of published studies, looking at the beneficial effects of oleuropein, there is limited clinical evidence focused on the benefits of this polyphenol as a nutraceutical product in humans, and many problems are still to be resolved about its bioavailability, bioaccessibility, and dosage. Thus, future clinical randomized trials are needed to establish the relation between the beneficial effects and the mechanisms of action occurring in the human body in response to the intake of oleuropein.

## 1. Introduction

The great progress of medical research has highly contributed to decreased mortality due to severe pathologies. But, on the other hand, a longer life expectancy has been associated with a greater incidence of illness and disability.

Non-communicable diseases (NCDs) are a group of long-lasting and slowly progressive chronic disorders [1]. The World Health Organization (WHO) recently reported that NCDs are the leading causes of death and disability for the general population, regardless of age, region, or gender [2]. NCDs have been deeply studied and some common key features have been identified; these include the intracellular presence of oxidative stress due to abnormal production of reactive oxidative species (ROS), inadequate antioxidant defense, and dysregulation of the autophagy pathway, responsible for the maintenance of cellular proteostasis [3]. Also inflammation is implicated in NCDs [4], since its level in an organism is closely related to cellular redox and an autophagic state [5,6].

Moreover, the health care costs associated with NCDs highlight the importance of finding new therapies for these pathological conditions, and it has been shown that healthy and equilibrated dietary patterns are useful in the prevention of NCDs [7].

The consumption of extra virgin olive oil (EVOO) is common in the Mediterranean Diet, which is largely known to have several health benefits and to increase longevity, as reported by the United Nations Educational Scientific and Cultural Organization (UNESCO) in 2010 [8,9]. As recently reported in the III International Conference on Virgin Olive Oil and Health Consensus Report, EVOO intake is also associated with reduced risk of most ageing-related diseases including cardiovascular and neurodegenerative diseases (CVD and NDD), and some types of cancer [10]. Initially, the beneficial properties of EVOO were attributed to functional components such as monounsaturated and polyunsaturated fatty acids (MUFAs and PUFAs), like oleic acid (55 to 83% of total fatty acid (FA)), the essential FA, linoleic acid (3 to 21% of total FA), and linolenic acid (0 to 1.5% of total FA). However, recent epidemiological and experimental studies also show that minor bioactive compounds, including phenolic alcohols, such as hydroxytyrosol (HT, 3,4-dihydroxyphenylethanol, 3,4-DHPEA) and tyrosol (*p*-hydroxyphenylethanol, *p*-HPEA), secoiridoid derivatives, phenolic acids, lignans, and flavonoids contribute to the beneficial effects of EVOO [11,12,13]. A high biophenol content confers a high stability to EVOOs, preventing EVOO autoxidation and contributing to a long shelf-life.

Oleuropein (Ole) is the major phenolic compound in the olive tree, *Olea europaea* L., and is particularly abundant in unprocessed olive fruit and leaves, with concentrations up to 140 mg g^−1^ on a dry matter basis in young olives [14], and 60–90 mg g^−1^ of dry matter in the leaves [15]. In *Olea europaea*, Ole, demethyloleuropein, ligstroside, and oleoside 11-methyl ester are abundant secoiridoids [16] whereas verbascoside [17] is the main hydroxycinnamic derivative of olives [18]

**Figure 1:**
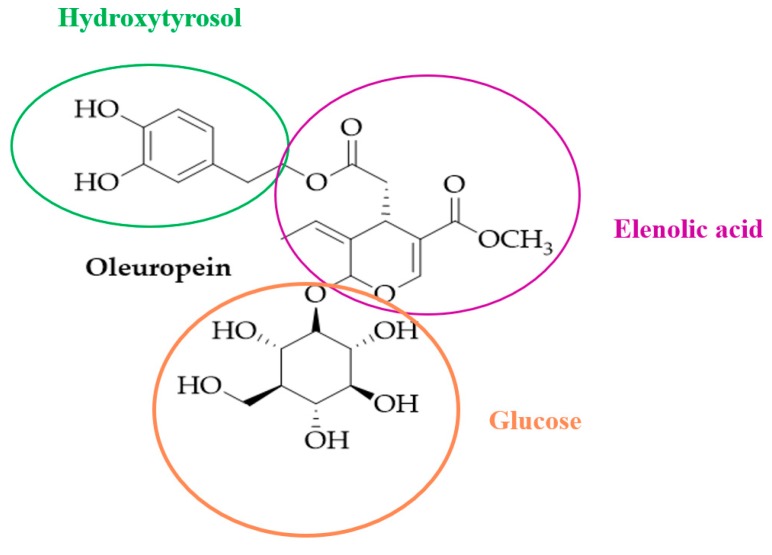
Chemical structure of oleuropein.

Ole belongs to the secoiridoids, which are abundant in *Oleaceas*, *Gentianales Cornale*s, as well as many other plants. Iridoids and secoiridoids are compounds that are usually glycosidically bound, and are produced from the secondary metabolism of terpenes, as precursors of various indole alkaloids. The secoiridoids in *Oleaceae* are usually derived from the oleoside type of glucosides (oleosides), which are characterized by an exocyclic 8,9-olefinic functionality, a combination of elenolic acid and a glucosidic residue [16]. Ole is an ester of elenolic acid and HT, and has a oleosidic skeleton that is common to the secoiridoid glucosides of *Oleaceae* (Figure 1)

Ole present in green olives, during the oil mechanical extraction process, is hydrolysed by the activity of endogenous β-glucosidases to form oleuropein aglycone (OleA), responsible for the bitter and pungent taste of EVOO. OleA together with other derivative secoiridoid species, such as the dialdehydic derivative of decarboxymethyl elenolic acid bound to either HT (3,4-dihydroxyphenylethanol-elenolic acid dialdehyde, 3,4-DHPEA-EDA, oleacein), or to tyrosol (*p*-hydroxyphenylethanol-elenolic acid dialdehyde, *p*-HPEA-EDA, oleocanthal), and ligstroside aglycone (*p*-HPEAEA), represents the minor polar compounds that determine the antioxidant capacity of EVOO. In recent years, oleocanthal and oleacein have attracted interest from the scientific community [19] due to their inflammatory effects. The first study was due to its similar structure to ibuprofen [20], the second for its ability to stimulate the expression of CD163, an anti-inflammatory gene [21]. The secoiridoid most extensively studied is OleA, whose content is dependent on the oil production process, as previously reported [22].

The relevance of bioactive components in EVOO has been strengthened by the European Food Safety Authority (EFSA), that in 2011, released a health claim [23,24] on the efficacy of oil phenols (5 mg/day per 20 g of EVOO, HT and OleA) in protecting low-densitiy lipoprotein (LDL) from oxidation, the initial event of atherosclerotic plaque formation. This is of interest because it is unique as a health claim that associates a specific dosage of a natural bioactive component of food with cardiovascular risk (LDL cholesterol oxidation) reduction [25].

Leaves of olive tree are a hystorical Mediterranean herbal drug, used as a traditional remedy for health promotion and a therapy for chronic conditions. The differences in Ole content depend on the cultivar, production area, and leaf tissue conditions (fresh, frozen, dried, or lyophilized). Commercial *Olea* leaf extracts, standardized in Ole content, were used to obtain food supplements with specific biological and biomedical properties [26].

Various methods have been developed for the qualitative and quantitative analysis of the occurrence of phenolic and secoiridoid compounds, from the simplest techniques, such as TLC [27], to the more sophisticated ones, such as reversed phase HPLC [22,28,29], GC-MS, FAMS, or TMS [30]. In the fruits, phenyl acids, flavonoids, and secoiridoids have been reported, the phenolic compounds representing 1–3% (*w/v*) [31]. In the leaves, 19% (*w/w*) is Ole and 1.8% (*w/w*) is flavonoids, of which 0.8% is luteolin 7-glucoside [15].

**Figure 2:**
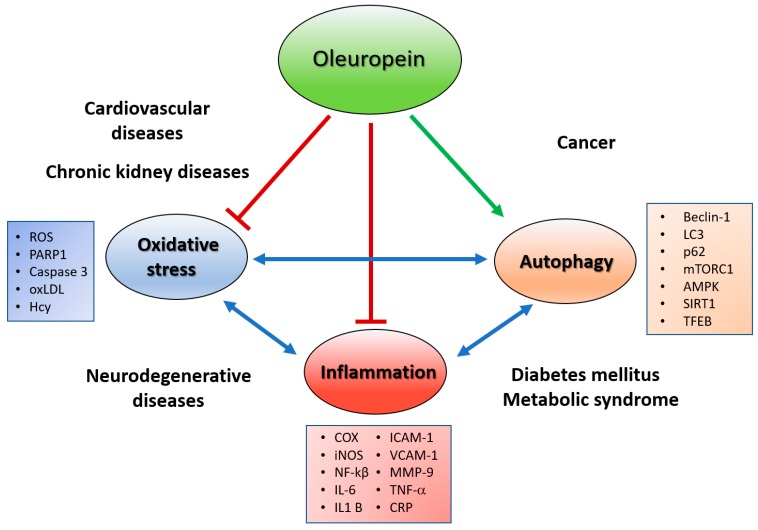
Effect of oleuropein on interplay between oxidative stress, autophaghy and inflammation in non-communicable diseases. AMPK, 5’ adenosine monophosphate-activated protein kinase; Beclin-1 autophagy-specific marker; COX, Cyclooxygenase; CRP, C Reactive Protein; Hcy, homocysteine; ICAM-1, Intercellular Adhesion Molecule 1; IL-1β, interleukin-1β; IL-6, interleukin-6; iNOS, inducible form of nitric oxide synthase; LC3 autophagy-specific marker; MMP-9, metalloproteinases-9; mTOR, mammalian target of rapamycin; NF-kB, Nuclear Factor Kappa-Light-Chain-Enhancer of Activated B Cells; oxLDL, oxidized low-density lipoprotein; p62 autophagy-specific marker; PARP1, Poly (ADP-ribose) polymerase; ROS, Reactive Oxygen Species; SIRT-1, NAD-dependent deacetylase sirtuin-1; TFEB, Transcription factor EB; TNF-α, tumour necrosis factor-α; VCAM-1, Vascular Cell Adhesion Molecule 1.

Recently, both oleuropein isoforms, glycosidic form and aglycones, have attracted scientific attention by virtue of their health benefits, such as antioxidant, anti-inflammatory, cardio- and neuro-protective, and anti-cancer effects. These pharmacological activities are mainly due to their putative radical scavenging features, due to the ortho-diphenolic group. Mechanistic studies indicate that these compounds are also able to act at different sites, interfering with protein function and gene expression, or modifying cellular pathways relevant to the NCDs pathological processes [25,32], suggesting that the actions of oleuropein in various disorders may result from shared molecular mechanisms. As reported above, dysregulated autophagy is a common feature of NCDs. This dysregulation seems to be due to increased oxidative stress, so, although these mechanisms are generally viewed as cell autonomous, recent evidence suggests an occurrence of an interplay between autophagy and oxidative stress that influences the inflammatory state of tissues, linked with NCD development [3] (Figure 2). The aim of this review is to collect and discuss the data available in the literature concerning the effect of oleuropein isoforms, Ole and OleA, in NCDs by their putative antioxidant and anti-inflammatory activities, but also through their other peculiar actions as autophagy inducers and amyloid fibril growth inhibitors. The last part of this review is dedicated to the anti-cancer effect of oleuropein and its ability to sensitize and potentiate the action of current therapies.

## 2. Oleuropein As an Antioxidant

The strong antioxidant properties of oleuropein is well known, and is shared with other phenols present in olive leaves and olive oil. In its chemical structure oleuropein contains an ortho-diphenolic group able to scavenge ROS through hydrogen donation, and to stabilize oxygen radicals with an intramolecular hydrogen bond. In particular, a *o*-diOH substitution confers a high antioxidant property, whereas single hydroxyl substitutions, e.g., tyrosol, provide none [33]. Ole in vitro can inhibit, in a dose-dependent manner, copper sulphate-induced oxidation of LDLs, assessed through a decrease in thiobarbituric acid-reacting substances and lipid peroxide by-product content [34,35]. In vivo, rabbits fed with an Ole-rich diet showed a higher serum antioxidant levels able to counteract LDL oxidation, and a reduction of total, free, and esterified cholesterol levels compared to animals receiving a standard diet [36]. Moreover, in humans, Visioli et al. [37] proved that Ole supplementation in healthy volunteers decreased, in a dose-dependent manner, the urinary excretion of 8-iso-PGF2α, suggesting a lower lipid peroxidation. A scavenging effect of Ole, similar to ascorbic acid and α-tocopherol, was shown in vitro against hypochlorous acid, a potent oxidant species produced in vivo by neutrophil myeloperoxidase at the site of inflammation [33], as well as against nitric oxide, as reported by De la Puerta et al. [38]. The first experimental evidence of the direct antioxidant cardioprotective effect of Ole against the post-ischemic oxidative burst after coronary occlusion, was reported by Manna et al. [39]. Using isolated rat hearts pretreated with Ole, subjected to global ischemia and then reperfused, they observed a decrease of creatine kinase and a reduced glutathione release in the perfusate, as well as a decrease of oxidized glutathione and in the lipid peroxidation level. Ole was also able to exert, in an indirect way, its antioxidant action by stimulating the expression of intracellular antioxidant enzymes *via* the activaction of Nuclear factor erythroid 2-related factor 2 (NrF2) transcription [40], as well as by increasing the level of non enzymatic antioxidants such as glutathione, α-tocoferol, β-carotene, and ascorbic acid [41,42,43].

## 3. Oleuropein As an Anti-Inflammatory and CVD Protective Agent

Inflammation is a crucial and defensive response induced by tissue damage or infection, and represents the “common soil” of multi-factorial diseases, playing a crucial role in promoting many disabling illnesses, such as atherosclerosis, diabetes mellitus, metabolic syndrome, cancer, chronic kidney diseases, and neurodegenerative diseases [44]. It can be divided into two types, acute and chronic. Chronic inflammation is correlated with the production of ROS that may cause oxidative damage and the depletion of antioxidants [45]. Macrophages, one of the main factors in the inflammatory response, produce ROS, but also pro-inflammatory cytokines and chemokines, including IL-1, IL-6, TNF-a, and IFN-γ. IL-6 seems to be the central mediator of the inflammatory response and an index of increased frailty [46,47,48,49]. Therefore, besides the release of several inflammatory cytokines or mediators, damaged tissues also release monocyte chemoattractant proteins (MCP-1), cyclooxygenase (COX), inducible form of nitric oxide synthase (iNOS), metalloproteinases (MMP), and adhesion molecules. In addition, nuclear factor Kappa β (NF-kβ) occupies a key upstream position in a complex signal transduction pathway, controlling the production of countless pro-inflammatory mediators [50].

In 2006, the PREDIMED trial first showed the anti-inflammatory effect of the Mediterranean Diet (MD) supplemented with EVOO for three months (compared with a low fat diet), through a remarkable decrease of serum C-Reactive Protein (CRP), IL-6, endothelial and monocytary adhesion molecules (ICAM-1 and VCAM-1), and chemokines, in a group of 722 partecipants [51]. Many other sub-studies of the PREDIMED trial have confirmed the anti-inflammatory properties of MD with EVOO by studying the changes in biomarkers associated with atherogenesis, such as peripheral blood mononuclear cell expression of cell surface inflammatory mediators (adhesion molecule and pro-inflammatory ligand CD40 expression on T lymphocytes and monocytes) or other molecules associated with systemic inflammation, such as those that induce the expression of adhesion molecules and activating NF- kB (TNFR60), or playing a role in T cell proliferation (TNFR80) [52,53,54]. Di Daniele et al. [55] demonstrated that the Italian MD in nephropatic patients could be a useful tool in the treatment of cardiovascular comorbidity related to renal dysfunction, causing a significant decrease in serum homocysteine (Hcy), dependant on a methylenetetrahydrofolate reductase genotype. In fact, Hcy, during the autoxidation process, induces impairment of the endothelium by producing ROS, with consequent involvement in atherosclerosis [56,57]. All these studies support that adherence to the MD with EVOO can modify inflammation, regardless of shared genetic and environmental factors.

In this context, many scientists have focused their attention on the possibility of using individual EVOO polyphenolic compounds like oleuropein, as a promising alternative anti-inflammatory agent, due to its capability to inhibit the synthesis of pro-inflammatory cytokines [58,59] and lipoxygenase activity [4], or to modulate inflammatory parameters [60].

Starting from in vitro results, Miles et al. [61] observed that Ole was the most powerful inhibitor of the production of IL-1β, compared with other phenols from EVOO, from human whole blood cells stimulated by lipopolysaccharide (LPS). Ryu S. et al. [62] verified that Ole was able to modulate the phenotype of LPS-activated murine macrophages, RAW 264.7, through the downregulation of key markers in inflammation pathways such as iNOS, COX-2, NFKB, and JNK, and of the two pro-inflammatory interleukins, IL-6 and IL-1β. The same authors also showed the efficacy of Ole in the reduction of LPS-induced NO in a zebrafish embryo model. 

OleA is able to modulate the tumor microenvironment, at least in part, through its anti-inflammatory properties, as reported by Margheri et al. [63]. Using “senescence-associated-secretory-phenotype” (SASP) fibroblasts, that show features of cancer-associated fibroblasts, they found that treatment with OleA decreased both the levels of SASP pro-angiogenic factors in the fibroblasts, and the release of the same in cell media, particularly IL-8. Interestingly, when endothelial progenitor cells and resident mature microvascular endothelial cells were exposed to these latter cell media, vasculogenesis and angiogenesis was inhibited by a decrease of MMPs and by the urokinase-type plasminogen activator, suggesting a mechanistic interpretation of the anti-angiogenic activities for cancer prevention by OleA.

The anti-inflammatory effect of Ole is better appreciated by studies using in vivo animal models. Giner et al. [64] demonstrated that Ole was able to ameliorate the symptoms of dextran sulfate sodium(DSS)-induced colitis in mice through the reduction of COX-2, iNOS, and MMP-9, and the suppression of p38 MAPK phosphorylation, which may be due to the up-regulation of annexin A1 [33]. Impellizzeri et al. [59] found that OleA could attenuate TNF-α and IL-1β production in a mouse model of carrageenan-induced pleurisy. The same pathways of TNF-α and IL-1β were also affected by Ole in a rat model of post-traumatic stress disorder [65], and in rats with spinal cord trauma [66]. Ole also showed its beneficial effect in an ovariectomy/inflammation experimental model of bone loss in rats, modulating parameters of inflammation, such us fibrinogen and spleen weight [67].

Recently, a study by Larussa T. et al. [68] demonstrated that the administration of OleA on colonic biopsies taken from ulcerative colitis patients led to a decrease of COX-2 and IL-17 levels, considerably reducing the inflammation of the colonic tissue. This evidence encourages the use of oleuropein as an inflammation-modulator.

### Lipid-Regulating, Anti-Hypertensive and Antidiabetic Effects of Oleuropein

CVD is a group of disorders affecting heart and/or blood vessels. CVD, including coronary heart disease (CHD), cerebrovascular disease, and peripheral arterial disease, are characterized by fatty deposits in the inner walls of the blood vessels supplying the heart and brain, that may cause an arrest of blood flow to these organs. The cardiovascular protective effect of oleuropein is supported by many in vivo animal studies and human clinical trials that showed, in addition to its antioxidant and anti-inflammatory properties, its lipid-lowering activity, anti-hypertensive, and hypoglycemic action [69,70,71].

Lockyer et al. [72] conducted a randomized, controlled trial on pre-hypertensive volunteers, who after an intake of Ole-enriched olive leaf extract for six weeks (136 mg Ole; 6 mg HT), showed significantly lower blood pressure (BP), plasma total cholesterol, LDL cholesterol, and triglycerids relative to the control, with a 5.76% reduction in coronary heart disease risk. Another trial on patients with stage-1 hypertension, showed that a daily dose of 2 × 500 mg of olive leaf extract (with 16–24% Ole) for four weeks, lowered systolic and diastolic BP with an effect comparable to that exerted by an effective dose (12.5–25 mg twice daily) of Captopril (the standard therapy for stage-1 hypertension), and reduced total plasma LDL and triglyceride levels. The authors concluded that the dual effect of olive leaf extract in lowering BP, probably due to angiotensin converting enzyme inhibition and calcium channel blocking activities, and improving lipid profiles, is advantageous for reducing the risk for CVD [73].

Insulin resistance is a systemic disorder, in which there is a reduced action of insulin despite an “hyperinsulinaemia” condition, that affects many organs, in particular the liver and adipose tissue, and leads to development of two NCDs, type 2 diabetes mellitus (T2DM) and metabolic syndrome, well known cardiovascular risk factors. Recent research has described the beneficial properties of OleA and Ole-enriched olive leaf extracts against T2DM, and other metabolic syndrome associated conditions. In particular, OleA prevents amylin aggregation into amyloid fibrils, whose pancreatic presence is considered one of the causes of the sufferance and functional impairment of insulin-secreting cells in T2DM (see 5: Oleuropein as anti–amiloid mean) [74] Therefore, many studies conducted in animal and cell models have reported that Ole has the property of decreasing blood glucose and cholesterol levels, and improving oral glucose tolerance and insulin sensitivity [41,75,76]. These findings were confirmed by human clinical trial results showing that treatment with Ole improved glucose homeostasis, reduced glycated hemoglobin and fasting insulin levels, suggesting a significant anti-diabetic effect [77,78,79]. Interestingly, in the context of these latter metabolic disorders, both characterized by insulin-resistance, de BocK et al. [78] showed a recovery of insulin sensitivity and pancreatic β-cell secretion capacity, in a group of overweight middle-aged men that received capsules of oleuropein-leaf extracts for 12 weeks, corroborating previous findings on the hypoglycemic effect of oleuropein [41,71,80].

Another disease highly associated with insulin resistance and the metabolic syndrome is non-alcoholic fatty liver disease (NAFLD), that affects about 25% of the world population, and the following non-alcoholic steatohepatitis (NASH). Research on cell and animal models have reported that oleuropein may counteract these conditions through different actions, including (i) an anti-lipidemic activity [81], (ii) protection and prevention of liver damage [82,83,84], and (iii) by interfering with signaling pathways involved in lipogenesis and in the onset of fatty liver disease [69]. Unfortunately, today these findings are not adequately supported by human studies, and remains unproven.

Therefore, in addition to the reported properties above, the ability of oleuropein to inhibit endothelial activation, monocyte cell adhesion and platelet aggregation within the concentration range expected after the nutritional intake from MD, suggest that oleuropein may also be considered an anti-atherogenic agent, reflecting its CVD protective activity [85,86,87,88,89]

## 4. Oleuropein As an Autophagy Inducer

Autophagy is a process by which the cells removes damaged organelles, malformed proteins or amyloid aggregate accumulation through lysosomal degradation. This is a process highly conserved and is required to maintain cellular homeostasis. It starts with the formation of a phagophore (that coincides with membrane isolation) that grows and terminates in auto-phagosome completion, which follows its fusion with lysosomes to form auto-phagolysosomes. Beclin-1 and LC3 are typical markers of autophagy activation, involved in the first steps of phagophore formation, while p62 participates in cargo recognition by lysosomes [90]. The target of rapamycin complex 1 (mTORC1) and the AMP-activated protein kinase (AMPK) are the stress sensors that control autophagy. However, while mTORC1 is an autophagy inhibitor activated by serum, nutrients, growth factors, etc., AMPK is instead an autophagy inducer, activated by low energy conditions and polyphenols.

Dysregulated autophagy is a common feature in NCDs implicated in NDD, metabolic syndrome, diabetes, CVDs, gastrointestinal diseases, and cancer [3]. As a master regulator of protein, lipid and carbohydrate metabolism, altered autophagy may concomitantly promote metabolic disorders and diseases associated with ageing, unhealthy diets, and inflammation. Indeed, knockout of the Atg7 gene in mice, an essential gene for autophagy, shows in vivo typical Parkinson’ disease (PD) features like Lewy bodies (LBs) formation, including endogenous synuclein and neuronal loss, as well as hepatomegaly with mutant hepatocytes showing accumulation of ubiquitin-positive aggregates [91,92]. A high fat-diet and genetically obese mice showed a decrease in autophagy flux, linked to elevated inflammatory gene expression [93,94,95]. Interestingly, autophagy seems to have a role in hypothalamic agouti-related peptide neurons in the regulation of food intake and energy balance, suggesting that the ability to regulate hypothalamic autophagy for modulating energy homeostasis may have implications in the development of new therapeutic options for obesity, and metabolic syndrome conditions [96]. Autophagic flux is also inhibited in pancreatic β-cells exposed to fatty acids, thus suppressing insulin secretion, a crucial factor for promoting T2DM to type-I diabetes conversion [97]. In the context of CDV, several studies show that autophagy might have beneficial or detrimental roles depending on the stage and type of the considered cardiovascular disease [4]. A beneficial function of autophagy has been observed in ischemia-reperfusion, cardiac hypertrophy, and atrial fibrillation. However, the majority of cardiac disorders suggests that autophagy may be a common cellular pathway that can be targeted for therapeutic gain, and the growing number of cardioprotective therapies affecting autophagic activity confirms this evidence [3,98,99]. Autophagy is also pivotal for intestinal homeostasis, appropriate intestinal immune responses, and anti-microbial protection, as well as neuronal and microglial functions [100]. In cancer cells, autophagy may exert either a tumor-promoting or tumor-suppressing effect [3]. Thus, it is still debated whether autophagy induction or inhibition may represent the most promising approach for future cancer treatments. Interestingly, cancer cells may also use autophagy as a resistance mechanism against chemotherapy [101]. In conclusion, autophagy is a key factor in the pathogenesis and regulation of various kinds of diseases, serving as a potential and effective target for their intervention. Therefore, the use of substances, such as polyphenols, that modulate autophagy and minimize the collateral effect, may be a valid therapeutic approach [102].

Some of the studies that contribute to demonstrating the healthful actions of oleuropein against pathologies involving autophagy dysfunction, acting as an autophagy enhancer through several mechanisms, and its potential use as a nutraceutical agent in several NCDs are summarized below

### 4.1. Oleuropein and NDDs

In our previous study performed in neuroblastoma cell lines, we found that OleA induced autophagy by activation of the Ca^2+^/Calmodulin Protein Kinase Kinase β (CaMKKβ)/AMPK/mTOR signalling axis. We proposed that OleA might induce autophagy through a Ca^2+^ increase in the cytoplasma from the endoplasmic reticulum that, in turn, activated Ca^2+^/CaMKKβ, and subsequently AMPK signaling. This complex facilitates mTORC1 inhibition and ULK1 activation to generate autophagic vacuole induction. We demonstrated that SH-SY5Y cells treated with 50 μM OleA showed an increased level of Beclin-1, critical for inducing autophagy, correlated with a biphasic elevation of the phosphorylation of residue Thr172 of AMPK [103]. The effects of OleA as an autophagy inducer have also been investigated in animal transgenic models. Grossi et al. [104], using a wildtype and TgCRND8 transgenic mouse model for human Aβ pathology, demonstrated that a diet supplemented with OleA restored the defective autophagic flux through an improvement of the fusion of lysosomes to autophagic vesicles, resulting in a remarkable cortex plaque reduction, and a recovery of the mice cognitive performance. They suggested that autophagy might be activated by inhibition of the mTOR pathway, reflected by the phosphorylation decrease of its target p70S6 protein kinase, shown in cell culture. These data indicate that autophagy may be considered as a strategic anti-amyloid mechanism, and suggest that OleA and/or its derivatives may exert their neuroprotective function in the brain, crossing the blood-brain barrier, acting as autophagy–related anti-amyolid agents, enhancing the clearance of Aβ plaque deposition. The cognitive recovery showed in these Alzheimer’s disease (AlzD) animal models supports the hypothesis that a diet supplementated with these polyphenols may have beneficial effects in slowing cognitive decline in patients with clinical signs of this disease.

Activation of NAD-dependent deacetylase sirtuin-1 (SIRT-1) is another mechanism through which OleA may modulate autophagy. SIRT1, a class III HDAC involved in the pathogenesis of several NCDs, deacetylates histones and non-histone proteins, such as transcription factors like p53, NF-κB, and FOXO, by transferring the acetyl group to NAD+. SIRT-1 influences autophagy directly (but also oxidative stress and apoptosis), via deacetylation of key components of this pathway. It showed a functional crosstalk with Poly (ADP-ribose) polymerase-1 (PARP-1) through NAD^+^ cofactor availability, and so any changes in levels of intracellular NAD^+^ and/or PARP-1 activity may influence SIRT-1 activity [105]. Luccarini et al. [106] showed that PARP-1 activation matched with a significant accumulation of PAR polymers in the cortex of TgCRND8 mice at the early (3.5 month) and intermediate (six month) stages of Aβ deposition. The same TgCRND8 mice fed with a supplementation of OleA showed a rescue of both PARP-1 activation, the accumulation of its product, and increased SIRT-1 expression. Moreover, OleA was able to reduce the rise of the apoptotic mediators phospho-NF-κB and phospho-p53.

### 4.2. Oleuropein and Cardioprotection

Miceli et al. [107] studied the effect of OleA as an autophagy enhancer in a cardiomyocyte model, characterized by autophagy dysfunction induced by oxidative stress due to a monoamine oxidase-A (MAO-A) overexpression. MAO-A is an isoform of FAD-dependent enzymes, that catalyzes oxidative deamination of catecholamines and serotonin in the heart, producing the corresponding aldehyde, H_2_O_2_, and ammonia. Previous studies have reported that MAO-A expression and activity increased in chronic cardiac diseases [108,109,110,111]. They found that OleA conferred cardioprotection, not simply by its antioxidant action, but through restoration of defective autophagic flux autophagy, reflected by auto-phagolysosome formation, measured by p62 and cathepsin-B levels increase, and the transcriptional factor EB (TFEB) activation and translocation to the nucleus. Translocation of TFEB to the nucleus modulated the transcription of autophagy genes prevented by MAO-A activation, reducing its transcriptional activity. They demonstrated that transcriptional regulation of autophagy by OleA was correlated with a significant cell death decrease, and to mitochondrial functionality recovery. These improvements disappeared after TFEB silencing, leading to the hypothesis that TFEB activaction was crucial for the protective effects of OleA against MAO-A-induced autophagy dysfunction. In addition, TFEB translocation and autophagy recovery induced by OleA did not affect ROS status in cardiomyocytes, further highlighting its peculiarity as an autophagy inducer.

### 4.3. Oleuropein and NAFLD

Using C57BL/6J mice fed a high-fat diet (HFD) for eight weeks, an animal model that well mimics human metabolic syndrome, with mice developing steatosis, metabolic and cardiovascular diseases, Porcu et al. [112] found an improvement in liver steatosis in mice fed the HFD with Ole that correlated with an increase of autophagy via the AMPK/ULK1 pathway, compared with animals fed with standard diet.

### 4.4. Oleuropein and Cancer

Recently HY et al. [113] found that inhibition of autophagy in a Triple-Negative Breast Cancer cell line promoted migration and invasion, as demonstrated by exposition with Hepatocyte Growth Factor (HGT), or 3-methyladenine, an inhibitor of autophagy. On the contrary, the co-treatment with HT or Ole significantly suppressed HGF or 3-MA induced cell migration and invasion, by reversing LC3 II/I and Beclin-1 downregulation, and p62 upregulation.

Increased autophagy seems to be a defensive mechanism against treatment with doxorubicin (DXR). Papachristodoulou et al. [114] demonstrated in prostate cancer cells, that Ole is capable of lowering the cytotoxic dose of DXR significantly, without losing its anti-proliferative effect, via an induction of autophagy (see: Section 7. Chemiotherapy potentiation by Oleuropein).

These findings contribute to demonstrate the healthful actions of oleuropein against pathologies involving autophagy dysfunction, acting as an autophagy enhancer throught different mechanisms, and suggest its potential use as a nutraceutical agent in several NCDs.

## 5. Oleuropein as Anti–Amyloid Tool

Many neurodegenerative pathologies, among which the most common are AlzD and PD, together with T2DM, are amyloid diseases (AD), and belong to the NCD group. In general, AD are diseases that are potentially fatal, defined by the occurrence of deposition of insoluble fibrillar polymeric material, grown from misfolded proteins (amyloid) in several organs. The core of these amyloids is made of unbranched polymeric fibrils of characteristic protein or peptides, typical for each disease, such as Aβ peptides for AlzD, α-synuclein for PD, amylin (hIAPP) for T2DM, and transthyretin (TTR) for familial amyloid cardiomyopathy [115,116,117]. Amyloidogenic proteins are characterized by β-sheet conformation, and share a common pathway of fibril formation. This latter is a complex process that involves the formation of an intermediate (soluble) oligomer form, following insoluble protofibril growth. Recently some authors have demonstrated that the cytotoxicity of different amyloidogenic proteins is due to soluble, intermediate oligomeric species, rather than to insoluble fibrillary amyloids [118]. Their cytotoxicity involves the disruption of calcium homeostasis, destabilization of membranes, ROS production, and apoptosis induction, all factors that determine cell suffering and death [119]. Interestingly, neurodegenerative diseases may extend outside the central nervous system (CNS), and can also involve the gastrointestinal tract (GI). Indeed, the same protein aggregates are present both in the enteric nervous system (ENS) and the CNS, leading to the hypothesis that the disease may start in the ENS and then spread retrogradely toward the CNS, or vice versa, and suggests that it may spread through a prion-like diffusion of misfolded protein accumulation, due to an imbalance between their production and clearance by autophagy systems [120].

So, the research of compounds interfering with aggregation of amyloid proteins is recognized as a valuable approach to build new therapeutic molecules. This is true expecially for AlzD, the most common form of dementia-related neurodegenerative disease among the elder people (aged 60 years and over), marked by a progressive decline in cognitive function and memory. Aggregates of Aβ peptides and neurofibrillary tangles of hyperphosphorylated tau proteins occurring in hypothalamic and cortical neurons are typical signs of this disease. PD is the second most common neurodegenerative disease, and is characterized by degeneration of dopaminergic neurons in the substantia nigra pars compacta due to deposition of intracellular inclusions known as LBs, the major component of which is α-synuclein. Although the clinical pathologies of these diseases have been described a long time ago, today there are drugs available only effective in reducing the symptoms of these diseases. The deposition of senile plaques and LBs in neuronal cells induces chronic stress, including oxidative stress and activation of microglial cells for the release of several pro-inflammatory cytokines, chemokines, and ROS, that are the major cause of these disorders [121].

OleA has been found to decrease toxic oligomers formation in vitro experiments of Aβ peptide and α-synuclein amyloid aggregation, as well as to promote fibril and plaque disaggregation [122,123,124]. These actions reflect its beneficial effects against amyloid toxicity to cultured cells [122] and in transgenic model organisms [125,126]. Using a simplified model of AlzD expressing human Aβ peptide, Diomede et al. [125] found that the larvae of a transgenic strain CL2006 of *Caenorhabditis elegans* fed with OleA, showed in the cytoplasm of muscle cells of the body wall, a reduction of Aβ plaque deposits, a lower content of toxic Aβ oligomers, a marked decrease of paralysis, and an increase of life expectancy compared with untreated animals.

hIAPP is a peptide hormone co-secreted with insulin by pancreatic β-cells, and fibrillar deposits of hIAPP amyloid aggregates in islets of Langerhans are a well known of T2DM. hIAPP aggregation together with oxidative stress (via NADPH-oxidase) leads to hIAPP toxicity, and plays a pivotal role in T2DM pathogenesis [127,128]. Rigacci et al. [74] showed that OleA occurrence, during the aggregation process, drove the formation of structurally different aggregates by hindering their binding with the membrane cells, resulting in a decrease of membrane damage, and protection of rat insulinoma cells against aggregate cytotoxicity, the main aspect responsible for cell sufferance. Similar results were obtained by Leri et al. [129] that extend the above finding to the OleA/TTR system, the latter is involved in a subset of familial or sporadic amyloid diseases including senile systemic amyloidosis (SSA), familial amyloid polyneuropathy and cardiomyopathy (FAP/FAC), for which no effective therapy has yet been found. This polyphenol was able to interfere with TTR fibril assembly and to promote mature fibril disruption. In this study, OleA protection against amyloid TTR toxicity, tested on in vitro HL-1 cells, resulted from stabilizing an oligomer-like intermediate that interacts with the plasma membrane without altering its integrity. In addition, OleA was also found to be able to disassemble pre-formed TTR mature fibrils into the same non-toxic oligomer-like intermediates [129].

Another interesting aspect of the anti-cytotoxic action of OleA, in the contest of amyloydosis, was reported by Leri et al. [130]. Using another amyloidogenic protein, a variant of human β2-microglobulin (β_2_m) [131,132,133] a 99 residue-long human protein belonging to the major histocompatibility complex class I (MHC I) associated with a familial form of systemic amyloidosis, they showed that it exhibited an enhanced amyloidogenic tendency to aggregate in vitro with respect to the wild-type protein. In this study, they found that OleA modified not only the conformational and biophysical properties of the amyloid fibrils, favoring the appearance of non-toxic aggregates, but also modified the cell bilayer surface properties, decreasing aggregate interaction with the plasma membrane of exposed cells, and enhancing cell resistance against the toxic effects of the aggregates.

All these data suggest a possible use of OleA as a novel and promising pharmacological tool, acting directly on amyloid formation *via* the protein self-assembly pathway, for prevention and therapy of systemic amyloidosis.

## 6. Oleuropein As an Anticancer Agent

Current protocols for cancer treatment are dependent on the condition of the tumor at time of diagnosis. If diagnosed early, the tumor mass may be removed by surgery, but if it has spread to lymph nodes, surgery will be more intensive, and chemotherapy and immunotherapy will likely be added to the treatment. Up-to-now, chemotherapy and immunotherapy represent a promising route for a more effective, life-saving cure for most human cancers. Despite advancements in these therapies, many patients with metastatic lesions still face a significant mortality risk. Furthermore, chemotherapy and immunotherapy may result in patient resistance, and generate host side effects. Therefore, new strategies that target cancer cells and also reduce resistance and patient side effects, may help the development of new treatments. Thus, the combination of conventional treatment with biological agents (so-called complementary therapy) may enhance the efficacy of treatment, and reduce drug resistance. In addition, complementary therapy may provide a reduction in side effects and improve the overall quality of life of patients during therapy.

**Figure 3:**
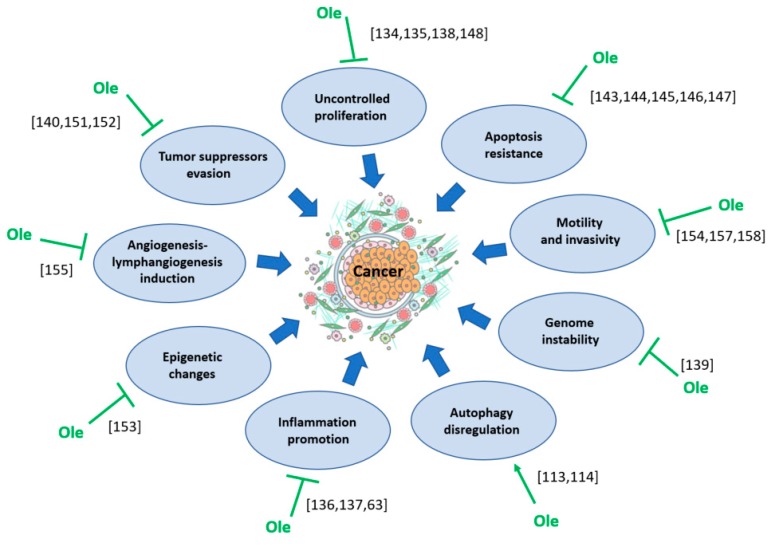
Effect of oleuropein (Ole) on the factors contributing to cancer development.

Oleuropein may contribute to therapy in several ways, including its inhibitory role in some crucial cancer cell activities, and these are summarized in the Figure 3.

### 6.1. Pro-Apoptotic and Anti-Proliferative Effects of Oleuropein

Bouallagui et al. [134], investigating Ole-enriched extract and its derivative HT, found a consistent inhibition of the proliferation of luminal breast cancer cells (MCF-7 cells), which were arrested in G0/G1 phase. The probable mechanism of growth-arrest was Cyclin D1 inhibition by these polyphenols. Han et al. [135] also reported in the same cancer cells, a blocking of the transition from G1 to S phase, caused by HT and OleA. Elamin et al. [136] was able to extend the use of Ole to the less responsive basal-like breast cancer cells (MDA-MB-231 cells), and demonstrated its ability to abrogate NF-kB expression, a well-known transcription factor involved in the control of many genes driving cancer development and progression, e.g., inflammation, immune reaction, proliferation, and apoptosis. This last finding was also confirmed by Liu et al. [137].

Further exposing BPH-1 normal prostate cells, androgen-sensitive LNCaP, and androgen-insensitive DU145 prostate cancer cells to Ole, promoted an anti-oxidant action in normal cells, whereas in cancer cells, it induced pro-oxidant and anti-proliferative effects, suggesting a quite cancer-specific effect [138].

It is also known that an Ole-enriched diet prevents azoxymethane-induced pre-neoplastic lesions of the colon, reducing dysplasia and DNA damage [139].

Cardeno et al. [140] showed that Ole and HT significantly inhibits HIF-1a and promotes the expression of p53 in HT-29 human colon adenocarcinoma cells, as a critical change to limit proliferation, and induce apoptosis.

Additional evidence suggests that one of the best inhibitors of human colon carcinoma cells is the HT oleate form, suggesting that long chain fatty acids, such oleate, may facilitate Ole activity [141].

Ole from Corregiola leaf extracts expresses a very high level of anti-proliferative activity on pancreatic cancer cells (MiaPaCa-2 cells), opening up a possibile treatment for one of the most aggressive human cancers.

Samara et al. [142] studied 51 analogs of Ole on several human cancer cells, and found that analog 24, non-toxic for normal cells, expresses the highest level of inhibitory activity in vitro (human colon cancer cells HCT-116, human cervical carcinoma cells HeLa, MCF-7 cells) as well as in vivo (B16-F10 mouse melanoma cells). Of a particular importance was the finding that Ole analog 24 expresses a promotion of natural immune responses, from Natural Killer cells and Limphokine-activated Killer cells.

Several authors showed Ole promoted apoptosis in cancer cells, like HeLa cells [143], HepG2 human hepatoma cells [144], SH-SY5Y human neuroblastoma cells [145], and HCT116 cells [146]. Taken together, the above researches provide significant insights into the contribution of Ole in treating cancer cells, disclosing several targets of its pro-apoptotic activity, such as activation of the JNK pathway, suppression of PI3K/AKT signaling, and activation of caspase-9 and 3 gene expression.

Additionally, pro-apoptotic activity of Ole has been seen against HL60 human promyelocytic leukemia cells [147].

### 6.2. Multiple Checkprotein of Transcription of Oleuropein

Further indications of the multiple targets of Ole activity on cancer cell machinery has emerged from the following contributions. Sirianni et al. [148] demonstrated that HT and Ole inhibit estrogen-dependent proliferation in MCF-7 cells, affecting extracellular-regulated kinases 1/2 (ERK 1/2) of the mitogen activating protein kinase family.

More-recently, Momtaz et al. [149] has shown the influence of Ole on melanoma. Melanoma is certainly one of the most aggressive skin cancers, due to its progression to drug resistance and its organ dissemination. Among the signalling pathways able to control melanoma development, janus kinase signal transducers and activators of transcription (JAKs/STATs) are the most critical, and polyphenols are able to target the most active, STAT3.

Furthermore, Ole was found to affect T-type Ca^2+^ channels altering the dynamics of intracellular Ca^2+^ in mesothelioma cancer cells. T-type calcium channels are low voltage channels, which open up when the membrane is depolarized, and calcium enters into various cells with different responses. On the other hand, mesothelioma is a cancer with a very poor prognosis, with an urgent need for new targeted therapies [150].

In MCF-7 cells, Ole exhibits an additional ability that extends its anti-cancer effects to the modulation of tumor suppressor genes, such as onco-miRNAs (miRNA-21 and miR-155) [151]. Researches by Tezcan G. et al. [152] serve to underline that Ole may modulate expression of miRNAs, miR-137, -145, and -153, in glioblastoma multiforme (GBM) cancer stem cells.

Bayat et al. [153], recently proved that Ole modulates epigenetic inhibiting histone deacetylases (HDAC2 and HDAC3). The use of HDAC inhibitors in cancer treatment represents a new approach to therapy, like reactivation of tumor suppressor genes. Although, this treatment causes side effects in the treated patients, Ole might have a role in some complementary therapies using HDAC inhibitors.

### 6.3. Other Biological Activities of Oleuropein

Ole was reported to express a potent inhibitory activity on tumor xenografts, disrupting actin filaments, thus abrogating proliferation, motility, and invasiveness [154]. The several cancer cell lines used in the experiments is an additional parameter of importance in this latter study (GMB cells, renal adenocarcinoma cells, breast cancer cells, melanoma cells, and colorectal adenocarcinoma cells).

Starting from the epidemiological observation that obesity is associated with an increased risk of developing many cancers, and metastatic dissemination represents the leading cause of mortality of cancer patients, a study by Song et al. [155] suggests that Ole inhibits tumor growth and lymph node metastasis in mouse melanoma cells, abrogating angiogenesis and lymphangiogenesis through the reduction of the peroxisome proliferator-activated receptor γ and the infiltration of M2 macrophages, both responsible for the secretion of angiogenesis and lymphangiogenesis inducers, VEGFA and D, respectively.

Evidence for the role of Ole in promoting the differentiation of K562 multipotent leukemia cells toward a monocyte lineage, was reported by Samet et al. [156]. Further anti-cancer/pro-apoptotic activity of Ole on leukemic cells, like HL60 human promyelocytic leukemia cells, was reported by Anter et al. [147].

The anticancer effect of Ole on glioma cells was characterized by a decrease of matrix MMP-2 and -9 activity, leading to a significant reduction in their ability to invade extracellular tissues [157]. In accord with this last observation, Ole was found to promote a reduction in the metastatic ability of breast cancer cells that was ascribed to a MMP-2 and -9 decrease, strengthened by a promotion of tissue inhibitors of MMP (TIMP) 1,3,4 [158]. It is known that a balance of MMP and TIMP regulates cancer cell invasion and dissemination.

## 7. Chemiotherapy Potentiation by Oleuropein

Due to the several anticancer properties of oleuropein, including anti-proliferative and pro-apoptosis activity, it was investigated whether it might represent an effective agent for complementary cancer therapy. In addition, it is well known that most anticancer drugs induce side-effects and toxicity in tumor-bearing patients, which oleuropein might be able to overwhelm.

A study reported by Papachristodoulou et al. [114] shows that co-treatment with doxorubicin and Ole affected cell proliferation of PC3 prostate cancer cells in an additive manner, even using a very low dose of doxorubicin. These authors also showed that the co-treatment did not modify cell cycle distribution and apoptosis of these cells in a significant way, but was able to induce a significant promotion of autophagy. Tumor cells are characterized by high energy use, even during starvation, when autophagy may sustain mitochondrial functions. Thus, the autophagy-dependence of cancer cells discloses a new therapeutic target for Ole, as reported above. Moreover, Ole has been shown to protect against the cardiotoxicity of doxorubicin [159]; this is an additional ability that further promotes the use of oleuropein in cancer co-treatments.

GBM is considered one of the most malignant human cancers, and therapy is up-to-now only palliative. GBM expresses a capillary network which contributes to tumor expansion and invasion, for which a targeted therapy using a monoclonal anti-VEGA antibody, bevacizumab, might be effective. However, this treatment often promotes an enhanced aggressive phenotype in resistant GBM cells. Tezcan et al. [160] were able to demonstrate that Ole synergistically increases bevacizumab’s anti-angiogenesis and anti-migration effects. Ole has the ability to promote the effects of bevacizumab, preventing VEGFA, MMP-2 and -9 activities. A large body of clinical and experimental evidence indicates a key role of PI3K/Akt/mTOR hyper-activation in GBM biology, which in turn, sustains cell metabolism by promoting protein synthesis and suppressing autophagy, the major protein degradation pathway [161]. In our previous study, we showed that in BRAF melanoma cells, Ole affected cell proliferation by downregulation of the pAKT/pS6 pathway [162]. Further studies and validation are required, but it is likely that Ole, through its mTOR inhibition and autophagy induction, may help in the development of new therapeutic strategies against GBM.

A further histotype of cancer with no effective treatment is hepatocellular carcinoma (HCC), which forms metastases and develops drug resistance very early. Thus, an effective and low-toxicity therapy is required. Sherif et al. [163] has demonstrated that Ole greatly potentiates the reduction of MMP-7 gene expression in HepG2 cells induced by cisplatin. This ability was found to be instrumental to reduce the cancer promoting ability of nerve growth factor (NGF) on HepG2 cells. Indeed, mature NGF is secreted from its precursor form, pro-NGF, through MMP-7 proteolytic cleavage, and exerts a pro-survival effect on HCC cells. The co-stimulation of Ole with cisplatin also enhances caspase-3 gene expression in these cells, potentiating their apoptotic rate.

Thereafter, our laboratory [162] demonstrated that Ole enhances chemotherapy of BRAF melanoma cells, by downregulating the pAKT/pS6 pathway. Of a particular significance, the finding that Ole was able to promote the death effect of Everolimus, a mTOR inhibitor, in Vemurafenib-resistant BRAF melanoma cells, points to a possibility for its use in treating resistant melanoma cells. OleA also contributed to the cytotoxic effect of dacarbazine against BRAF melanoma cells. As in resistant melanoma cells, exposure to OleA was found to reverse trastuzumab resistance in HER2-overexpressing breast cancer cells [164].

In addition to chemotherapy, Ole has been proved to enhance the radiation sensitivity of nasopharyngeal carcinoma cells repressing mRNA-519d [165].

On the whole, oleuropein has attracted the attention of several researchers, due to its ability to discriminate between cancer and normal cells, inhibit proliferation, and promote apoptosis in several tumors, including those tumors which are considered the most aggressive, such as mesothelioma, GBM and melanoma. Finally, a real potentiating effect of oleuropein on standard chemotherapy has been demonstrated.

## 8. Bioavailability of Oleuropein

The beneficial effects of olive leaves or different preparations (e.g., infusions, extracts) have been known since ancient times, and have been used as traditional herbal remedies for the treatment of many diseases (such as diabetes mellitus, artherial hypertension, and bronchial asthma), or to alleviate their symptoms. Olive leaves can be a good source for the development of new potentially functional foods that may contribute, through basic nutrition, to optimal health conditions reducing the risk of NCDs. Therefore, special attention is paid to the recovery, recycling, and upgrading of food waste and by-products [166,167,168,169]. Ole is the major constituent of the secoiridoid family in olive leaves. HT is the primary metabolite of Ole/OleA, and shares some of the above described biological properties, but possesses a greater antioxidant capacity. Although this feature increases the importance of HT, obtaining it in a synthetic form is expensive, so methods using Ole as a source of HT production, have been developed [170,171,172].

The possibility that these biophenols may exert their biological effects depends on the probability of reaching key molecular targets in human tissues at a sufficient dose, which is dependant to their metabolism and bioavailability. However, the data on the metabolism of oleuropein from EVOO or olive leaves in humans are poor, and often the results on the level and the form found in plasma, and/or excreted in urine, are conflicting [173,174,175]. This discrepancy may be explained by the fact that oleuropein bioavailability is influenced by several factors, such as the route of administration, genotype, age, sex, interaction with food, and by the different extraction processes and analytical methods used [176]. A recent human trial showed that oral Ole ingestion is resistant to the acidic conditions of the stomach, and it is rapidly absorbed (55–60%) in the intestinal tract, reaching a maximum plasma concentration (23–30 min, depending on the preparation, liquid vs. capsule) earlier than conjugated metabolites of HT, glucuronidated and sulfated (at 64–93 min), that made up 96–99% of the Ole phenolic metabolites detected in plasma and urine after intake [177]. These data suggest a potential complete metabolization of Ole to HT, and other degradation products. The efficacy in vivo of these compounds regarding their absorption and metabolism kinetics once ingested, should be checked. In fact, the major criticism of the in vitro studies using these molecules is that the doses used are at greater concentrations (µmol/L–mmol/L) compared to the metabolite concentrations measured in plasma, which are only at the nmol/L concentration [178]. Therefore, delivery systems have been developed based on the esterification/lipophilisation and encapsulation of phenolic compounds, or using the creation of liposomes and/or nanoparticles of bioactive compounds, to increase their bioavailability and bioaccessibility [179,180,181,182].

Recently, much attention has been given to the gut microbiota, considered as a metabolic ‘‘organ” which impacts host nutrition, and may influence the bioavailability and bioaccessibility of olive phenolic compounds *via* biotransformation into other active substances, which have interesting beneficial health properties in bowel diseases [166,183]. Mosele et al. [184], in an in vitro model experiment, observed that Ole was rapidly deglycosylated during 6 h of incubation with human fecal microbiota, becoming OleA; the latter was degradated into elenolic acid and HT by microbial esterase activity, until it disappeared after 48 h. On the contrary, HT constantly increased during the same fermentation period. This finding was confirmed by the same authors in an in vivo study, that showed that after intake of phenol-rich olive oil for three weeks, the concentration of free HT was significantly increased in the faeces of all the participants in the study. Other reserches have shown that the conversion of Ole into HT was performed by lactic acid bacteria, in particular by *Lactobacillus plantarum* [185], and recently some authors developed oral granules for the co-delivery of *L. plantarum* and a standardized olive leaf extract (Phenolea^®^Active F, PhenoFarm s.r.L, Rome, Italy), in order to foster Ole metabolism and provide high levels of HT [171].

## 9. Conclusions

The evidence presented in this review demonstrates the several biological activities of oleuropein including antioxidant, anti-inflammatory and anticancer properties. Several epidemiological studies have reported a strict association between a diet rich in this polyphenol and the prevention of several NCDs, that are among the main causes of morbidity and mortality in the world. In conclusion, several in vitro and in vivo studies have demonstrated the ability of oleuropein (and its derivatives) to counteract oxidative stress and inflammation, to modulate the autophagy pathway, as well as to interfere in the amyloid aggregation process, suggesting its use, not only in the prevention, but also as a complementary therapy of some diseases. Despite the low bioavailabilty of oleuropein, some clinical trials reported several beneficial effects after administration of this compound, confirming the results obtained in vitro and in vivo studies. The effective daily dose of oleuropein to be administered in humans to achieve a theraputic effect is not known, but clinical and experimental evidence suggest that regular intake of this compound can be effective in the long term, representing a continuous low-intensity stimulus to the cellular defence against NCDs [186].
